# Mitochondrial bioenergetic is impaired in Monocarboxylate transporter 1 deficiency: a new clinical case and review of the literature

**DOI:** 10.1186/s13023-022-02389-4

**Published:** 2022-06-21

**Authors:** Sinziana Stanescu, Irene Bravo-Alonso, Amaya Belanger-Quintana, Belen Pérez, Montserrat Medina-Diaz, Pedro Ruiz-Sala, Nathaly Paola Flores, Raquel Buenache, Francisco Arrieta, Pilar Rodríguez-Pombo

**Affiliations:** 1 Unidad de Enfermedades Metabólicas, Hospital Universitario Ramón y Cajal, IRYCIS, Crta de Colmenar Viejo, km 9,100, 28034 Madrid, Spain; 2 Centro de Diagnóstico de Enfermedades Moleculares, Centro de Biología Molecular Severo Ochoa, UAM-CSIC, CIBERER, IdiPAZ, C/Francisco Tomás y Valiente, 7, 28049 Madrid, Spain; 3 Department of Neuroradiology, Hospital Universitario Ramón y Cajal, IRYCIS, Crta de Colmenar Viejo, km 9,100, 28034 Madrid, Spain; 4 Centro de Diagnóstico de Enfermedades Moleculares, Centro de Biología Molecular, Universidad Autónoma de Madrid, CIBERER, IdiPAZ, C/Francisco Tomás y Valiente, 7, 28049 Madrid, Spain; 5 Paediatric Department, Hospital General La Mancha Centro, Av. Constitución, 3, 13600 Alcázar de San Juan, Ciudad Real Spain; 6 Neuropediatric Department, Hospital Universitario Ramón y Cajal, IRYCIS, Crta de Colmenar Viejo, km 9,100, 28034 Madrid, Spain; 7 Unidad de Enfermedades Metabólicas, Hospital Universitario Ramón y Cajal, IRYCIS, CIBER-OBN, Crta de Colmenar Viejo, km 9,100, 28034 Madrid, Spain

**Keywords:** Monocarboxylate transporter 1, Recurrent acidosis, Mitochondrial dysfunction, Psychomotor delay, White mater alterations, Corpus callosum agenesia

## Abstract

**Background:**

Monocarboxylate transporter 1 (MCT1) deficiency has recently been described as a rare cause of recurrent ketosis, the result of impaired ketone utilization in extrahepatic tissues. To date, only six patients with this condition have been identified, and clinical and biochemical details remain incomplete.

**Results:**

The present work reports a patient suffering from severe, recurrent episodes of metabolic acidosis and psychomotor delay, showing a pathogenic loss-of-function variation c.747_750del in homozygosity in *SLC16A1* (which codes for MCT1). Persistent ketotic and lactic acidosis was accompanied by an abnormal excretion of organic acids related to redox balance disturbances. Together with an altered bioenergetic profile detected in patient-derived fibroblasts, this suggests possible mitochondrial dysfunction. Brain MRI revealed extensive, diffuse bilateral, symmetric signal alterations for the subcortical white matter and basal ganglia, together with corpus callosum agenesia.

**Conclusions:**

These findings suggest that the clinical spectrum of MCT1 deficiency not only involves recurrent atacks of ketoacidosis, but may also cause lactic acidosis and neuromotor delay with a distinctive neuroimaging pattern including agenesis of corpus callosum and other brain signal alterations.

**Supplementary Information:**

The online version contains supplementary material available at 10.1186/s13023-022-02389-4.

## Background

The monocarboxylate transporter (MCT) protein family is a diverse group of transmembrane proteins encoded by the *SLC16* gene family. These genes are distributed across all mammalian species and are involved in many metabolic pathways, including those used by the brain, skeletal muscle, heart and tumor cells [1]. Of the 14 members of the *SLC16* family, MCT1 and MCT4 have been shown to undertake proton-coupled symport of important monocarboxylate metabolites, such as pyruvate, L-lactate and ketone bodies (acetoacetate and 3-hydroxy butyrate) [1–3].

MCT1, which is encoded by *SLC16A1*, is responsible for the import of monocarboxylates across the plasma membrane. It is expressed in nearly all human tissues, the most notable exception being the pancreatic β-cells. Genetic variations in *SLC16A1* have been associated with different human pathological conditions. Dominant gain-of-function mutations in the promoter region of *SLC16A1* provoking the abnormal expression of MCT1 in pancreatic β-cells have been identified in patients suffering from exercise-induced hyperinsulinemic hypoglycemia (MIM#610021), a clinical condition associated with dysregulated insulin secretion in states of low plasma glucose during anaerobic exercise [4].

Deficient MCT1 activity due to inactivating nucleotide changes in *SLC16A1* has been recorded in patients suffering from a ketone metabolism disorder caused by deficient ketone delivery to the extrahepatic tissues (MIM#616095). The first clinical description was made in 2014 in patients with severe recurrent episodes of ketosis, decreased consciousness and dehydration triggered by concurrent infections or fasting [5]. Since then, other authors have reported problems in biallelic homozygous patients, who, on top of recurrent ketoacidosis, also suffer from epilepsy and developmental delay, and who show neuroimaging anomalies of the subcortical white matter and basal ganglia, and/or agenesis of the corpus callosum [6, 7]. Interestingly, monoallelic carriers of variants in *SLC16A1* can also result in clinical symptoms such as cyclic vomiting and ketoacidosis [5, 8, 9]. In summary, MCT1 deficiency has been described in both monoallelic carriers with a potentially milder phenotype, and biallelic patients suffering a severe course of the disease. However, a combination of pathogenic variants in heterozygosity with other genetic and/or environmental factors has been suggested to contribute towards patients' clinical and biochemical phenotypes [8].

The present work reports the clinical and biochemical findings for a new patient with a homozygous mutation in *SLC16A1*. To understand the underlying pathophysiology involved, bioenergetic analyses were performed in patient-derived fibroblasts. The results clearly suggest a mitochondrial dysfunction not previously described.

## Materials and methods

Written informed consent to be include in this study was provided by the patient’s parents. The study protocol adhered to the Declaration of Helsinki, and the protocol was approved by the Ethics Committee of *Universidad Autónoma de Madrid* (CEI-105-2052).

*Targeted analysis of metabolites* Organic acids in liquid urine samples were determined as trimethylsilyl derivatives by GC–MS after urease treatment and ethyl acetate liquid–liquid extraction without oximation [10].

### Genetic analysis

Genomic DNA was extracted from peripheral blood using the MagnaPure system (Roche Applied Science, Indianapolis, IN, USA) and subjected to massive parallel sequencing using the Nextera DNA Exome Kit (Illumina, San Diego, CA, USA) for whole exome sequencing (WES). Variant calling was undertaken using DNAnexus (Mountain View, CA, USA) and an in-house bioinformatic pipeline. Overall mean exon coverage was 78×, with a ≥ 20× base coverage of 84%.

Variants were initially filtered based on their predicted pathogenicity and minor allele frequency (< 0.005). All genes with variants that survived this filtering were then screened using virtual panels based on phenotypic importance according to human phenotype ontology terms (HPO) [11] or the MitoCarta database [12].

Filtering also included the presence of gene variants previously annotated in the Human Gene Mutation Database (HGMD^®^ Professional 2021.2) [13]. The potential pathogenicity of the selected variants was assessed using the VarSome web platform [14] which takes into account data from the dbSNP, ClinVar, gnomAD, RefSeq, Ensembl, dbNSFP, Gerp, Kaviar, CIViC databases, and runs the DANN, dbNSFP, FATHMM, MetaLR, MetaSVM, Mutation Assessor, PROVEAN, GERP, LRT and MutationTaster-prediction programs.

### Cell culture

Healthy and patient-derived dermal fibroblasts obtained from a skin biopsy (taken with informed consent) were grown in minimal essential medium (MEM) supplemented with 1% glutamine, 10% foetal bovine serum (FBS), and antibiotics, following standard conditions. As healthy control-derived fibroblasts we used the cell lines CC2509 (Lonza, Basle, Switzerland) and GM8680 (Coriell Institute for Medical Research, Camden, NJ, USA). Most of experiments were accomplished when fibroblasts were at 80% confluence.

### Mitochondrial function

The cellular oxygen consumption rate (OCR) was measured in an XF24 Extracellular Flux Analyzer (Seahorse Bioscience, Izasa Scientific) according to [15], except that cells were raised in the same MEM as above but with galactose (1 g/L) instead of glucose during 24 h. After taking an OCR baseline measurement, oligomycin, carbonyl cyanide-4-(trifluoromethoxy) phenylhydrazone (FCCP), rotenone and antimycin A solutions were sequentially added to each well to reach final working concentrations 6 μM, 20 μM, 1 μM and 1 μM respectively. Each condition was measured three times every 7 min as indicated by the commercial kit. Basal respiration was measured without substrates. Oxygen consumption coupled to ATP production (ATP-linked) was calculated as the difference between basal respiration and the proton leak state determined after the addition of oligomycin. Maximum respiration was measured by stepwise 20 μM titrations of FCCP and inhibition by rotenone and antimycin. Spare capacity was calculated as the difference between maximum and basal respiration. Data are expressed as the OCR in picomoles per minute for 60,000 cells.

### Morphometric analysis of mitochondria

Morphometric analyses of mitochondria were made using a Jeol JEM-1010 (Jeol Ltd, Tokyo, Japan) electron microscope operating at 80 kVas, as described elsewhere [16]. Images were recorded with a 4 k CMOS F416 camera (TVIPS, Gauting, Germany). The aspect ratio was defined as the major axis/minor axis [17] and was measured in at least 50 randomly selected mitochondria. A minimum aspect ratio of 1 corresponds to a perfect circle.

### Mitochondrial isolation and Western blotting

Mitochondria were isolated using the hypotonic swelling procedure [18]. These mitochondria were then denatured in Laemmli buffer for 5 min at 50 °C, and extracts subjected to SDS-PAGE separation and Western blotting [15]. For native PAGE analysis, protein samples were prepared following the indications provided with the NativePAGE™ Novex^®^ Bis–Tris Gel System (Invitrogen, Carlsbad, CA, USA). Fifteen micrograms of mitochondrial sample were resuspended in a medium with 2% digitonine, and then loaded onto 3–12% NativePAGE™ Novex gel. The primary polyclonal antibodies used were: anti-total OxPhos (NDUFB8-CI, SDHB-CII, UQCRC2-CIII, MTCOI-CIV and ATP5A-CIV) (ab110413; Abcam, Cambridge, UK), anti-NDUFA9 (ab14705, Abcam), anti-MTCOI (ab14705, Abcam) and anti-ATP5A (ab14748, Abcam). Anti-citrate synthase (C5498, Sigma Aldrich) and anti-SDHA (ab14715, Abcam) were used as loading controls. Films were scanned and densitometered using the BioRad GS-900 scanner and Image Lab 5.2 software.

### Statistical analysis

Values are expressed as means ± SEM of ‘n’ independently performed experiments in cultured cells. Diferences between means were examined using the Student t test. Significance was set at *p* < 0.05. All calculations were performed using GraphPad Prism 6 (GraphPad Software, La Jolla, CA, USA).

## Results

### Case report

The present patient, a female infant, is the second child of non-consanguineous Moroccan parents. There was no relevant family history; her oldest brother was diagnosed at the age of 8 years old of type I diabetes mellitus. Corpus callosum agenesis (HP:0001274) was detected during prenatal monitoring and later confirmed by clinical assessment. Her birth and perinatal period were normal. At the age of 4 months she had an episode of severe acidosis (pH 6.7) (HP:0001942) during a respiratory infection. Plasma 3-hydroxy butyrate and lactate levels were increased at 2.3 mmol/L (HP:0033419) and 7.0 mmol/L (HP:0003128) respectively; ammonia, glucose, and liver function remained normal. She was diagnosed with sepsis, treated with antibiotics and fluid replacement, and made a full recovery. No further metabolic studies were performed. At the age of 15 months she suffered two more episodes of severe acidosis (pH 7.0 on both occasions) associated with dehydration and reduced consciousness (HP:0007185) following vomiting (HP:0002013) and fasting. She was admitted to a regional hospital and her condition improved with continuous infusion of dextrose. Her plasma lactate was mildly elevated (2.6 mmol/L) and her glucose slightly low (2.5 mmol/l) (HP:0001943), but no ketone body analysis was performed during the initial check-up.

After these episodes she was transferred to our metabolic unit for further investigation. In her basal state, she persistently maintained mildly elevated lactate plasma levels (2.3–2.5 mmol/L); urine gas chromatography showed increased excretion of ketone bodies (HP:0410175), lactate, 2-hydroxy butyrate and 3-hydroxy isovalerate, all compatible with an underlying mitochondrial disorder (see Table 1). Slight elevation of plasma alanine (Ala) was detected in one determination, but it was not sustained (see Table 1). Lactate and amino acids levels in cerebrospinal fluid were normal in basal state [lactate: 1.67 mM, Ala: 30 μmol/L (NV: 30 ± 11)].

**Table 1:** Metabolic study at different times in the patient's life and after 18 h of fasting

Age	7 Months (no acidosis)	10 Months (no acidosis)	15 Months (no acidosis)	15 Months, 18 h of fasting
*Urine organic acids (mmol/mol creatinine)*				
3-Hydroxy butyrate (NV: 2–17)	158	1114	284	15,471
Acetoacetate (NV: 0–7)	117	792	20	9342
Lactate (NV: 5–113)	9312	359	28	23,846
2-Hydroxy butyrate(NV: 0–4)	380	273	15	11,944
3-Hydroxy isovalerate (NV: 1–40)	56	56	19	315
*Plasma amino acids (μmol/L)*				
Alanine (NV: 297 ± 96)	435	230	329	124
Others	Normal	Normal	Normal	Elevation of BCAA due to severe ketosis
*Plasma acylcarnitines μmol/L*				
	Normal	Normal	Normal	Mild elevation of C12–C14 due to ketosis

*NV* normal value, *C12* dodecanoylcarnitine, *C14* myristoylcarnitine, *BCAA* branched-chain amino acids

A fasting test was performed and after 18 h the patient showed hypoglycemia (2.2 mmol/L) with ketosis (3-hydroxy butyrate: 5.2 mmol/L) and significant metabolic acidosis with a pH of 7.28, a plasma bicarbonate concentration of 7.5 mmol/L, and base excess of − 17.5 mmol/L. She was treated with continuous infusion of dextrose and bicarbonate supplements. Metabolomic studies made during her fasting revealed the massive excretion of 3-hydroxy butyrate, acetoacetate, lactate, 2-hydroxy butyrate and 3-hydroxyisovalerate (see Table 1).

**Fig. 1:**
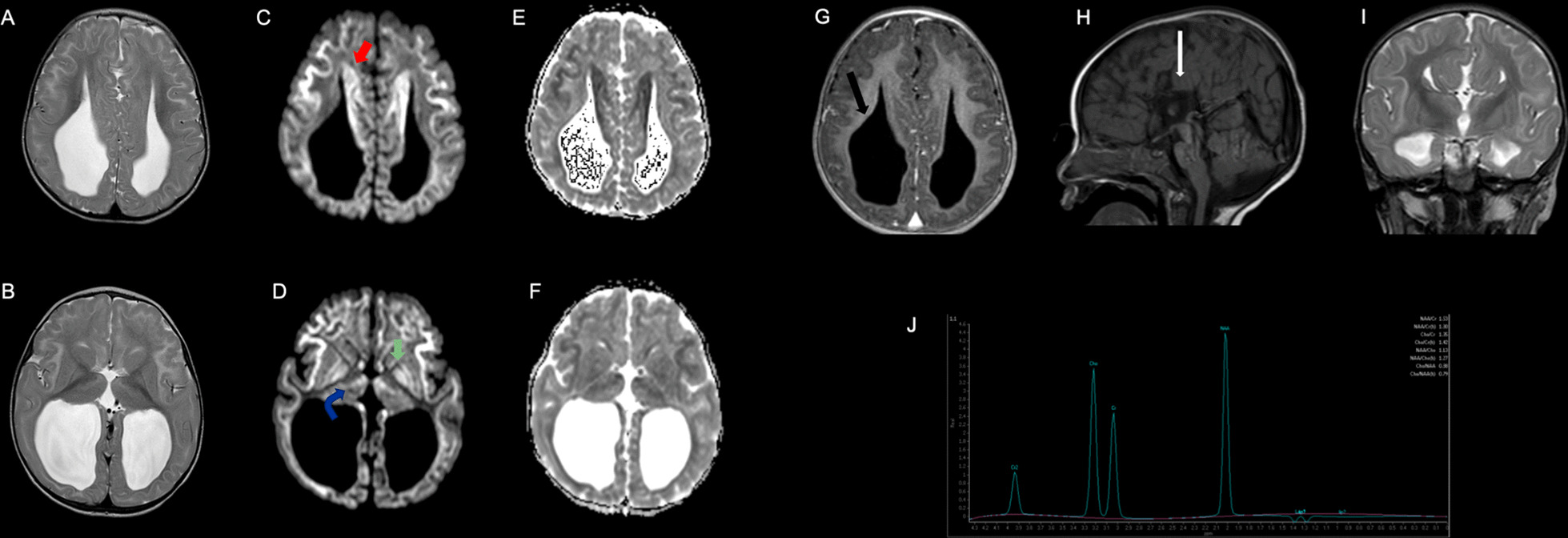
Brain MRI of the present patient at 23 months of age, showing extensive bilateral symmetric, anomalous signals for the subcortical white matter and basal ganglia, and corpus callosum agenesia

A brain MRI performed at the age of 23 months revealed diffuse, bilateral, symmetric signal alterations for the subcortical supratentorial white matter, with an anteroposterior gradient, especially significant in the frontal and insular areas. These alterations consisted of hyperintensity in the T2-weighted sequence (HP:003090) (Fig. 1A, B) affecting the U fibers, a hypersignal in the diffusion-weighted sequence (Fig. 1C, D), and low apparent diffusion coefficient (ADC) values (Fig. 1E, F). This same pattern of signal alteration was seen in the Probst bands (Fig. 1, red arrow), in the anterosuperior and medial regions of the thalamus (Fig. 1, blue arrow), and in both putamens, especially in the globus pallidus (Fig. 1, green arrow). No enhancement was observed in the T1-weighted sequence after intravenous contrast administration (Fig. 1G). She also showed complete agenesis of the corpus callosum (HP:0001274) (Fig. 1, white arrow) with colpocephaly (HP:0030048) (Fig. 1, black arrow) and marked loss of volume of the predominantly posterior white matter (Fig. 1G–I). Single voxel, long TE proton MRI spectroscopy showed no pathological peak for lactate (Fig. 1J).

Frequent feeding (maximum fasts of 6 h) and adequate energy intake were recommended during episodes of illness. She has had no further acute episodes of acidosis and/or ketosis, and her current plasma lactate levels are normal. At the age of 6 years, clinical examination revealed moderate psychomotor delay (HP:0001263): she has an unsteady gait (HP:0002317) due to axial hypotonia (HP:0008936) and her language skills are poor (HP:0002463).

Although a ketolytic defect was the main clinical suspicion, she was included in a genetic testing program designed for patients showing persistent lactic acidosis, and was classified as having a *probable mitochondrial disorder.* The mitochondrial disease score was based on the sum of her clinical and biochemical symptoms according to the Nijmegen system, modified by Morava et al. [19].

### Mitochondrial function

**Fig. 2:**
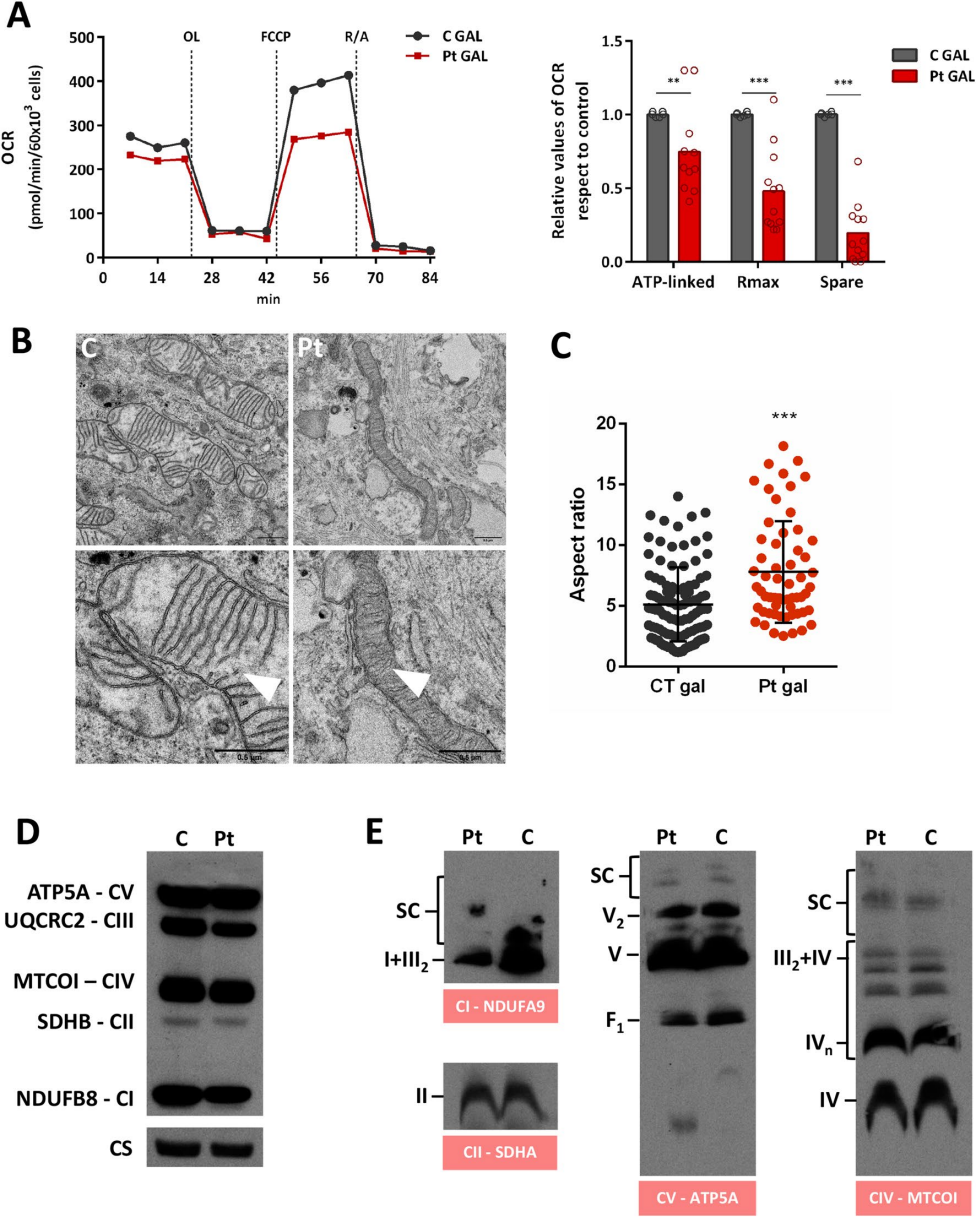
**A** Oxygen consumption rates of control (**C**) and patient-derived (Pt) fibroblasts measured in MEM with galactose (1 g/L) (GAL) instead of glucose, and upon the subsequent addition of (a) 6 μM oligomycin (OL), (b) 20 μM FCCP, (c) 1 μM rotenone and 1 μM antimycin (R/A). Oxygen consumption coupled to ATP production (ATP-linked), maximum respiration (Rmax) and spare capacity (Spare) were calculated for each situation. Results are expressed as fold-change over the control concentrations and are the mean ± SD of 3–5 wells from three independent experiments represented by circles in the bar graph. Control values are the means of the two control cell lines (CC2509 and GM8680). **B** Electron microscopy images showing defects in mitochondrial ultrastructure and cristae organization (white arrows) in patient-derived fibroblasts grown in MEM with galactose. Mitochondrial length was determined in control (**C**) and patient-derived (Pt) fibroblasts. **C** Mitochondrial enlargement is expressed as the aspect ratio (major/minor mitochondrial axis ratio). Measurements were made for at least 50 mitochondria. Statistical analysis were performed using GraphPad Prims software. Student t test (**p < 0.01; ***p < 0.001). **D** Western blot of Oxphos (SDS-PAGE-separated) from purified mitochondria. **E** Blue native gel, staining of complex I (NDUFA9), complex II (SDHA), complex IV (MTCOI) and complex V (ATP5A) in purified mitochondria CI: complex I; CII: complex II; CIII: complex III; CIV: complex IV; CV: complex V; CS: Citrate Synthase; SC: supercomplexes

To assess the mitochondrial functionality, we accomplished a real-time oxygen consumption test, visualised the mitochondrial ultrastructure and evaluated the presence of OxPhos proteins, both individually or ensembled in respiratory complexes. To force a mitochondrial synthesis of ATP, controls and patient fibroblasts were grown in galactose-media. The Seahorse data revealed a robust reduction in maximum respiration and spare capacity comparison to controls, indicating diminished cell capacity to respond to stress stimuli or higher metabolic demands (Fig. 2A). In concordance with these results, we observed an altered mitochondria ultrastructure of patient-derived fibroblasts with disrupted cristae structure (Fig. 2B—white arrows) and significant increases in the number of elongated mitochondria (Fig. 2C), all compatible with an energetic challenge. Mitochondria from control cells showed a characteristic zebra-striped appearance.

Finally, Western blotting analysis of OxPhos proteins and respiratory complex in mitochondrial extracts revealed a notable reduction in the fully assembled CI (Fig. 2E) of the patient compared to control analyzed by Blue Native PAGE. No major changes were detected in either other complexed or individual OxPhos proteins visualized by SDS-PAGE (Fig. 2D).

### Genetic analysis

In an early genetic analysis of the clinical exome, the change NM_003051.4:c.747_750del (p.Asn250Serfs*5) was identified in homozygous fashion in *SLC16A1* [15]. This variant had been previously described in monoallelic carriers patients with ketoacidosis and massive ketonuria [5]. Sanger sequencing confirmed the presence of this change in heterozygous fashion in the patient's healthy progenitors and one sibling. According to the literature, the presence of c.747_750del explains the persistent ketoacidosis, but not the mitochondrial dysfunction signature. Other possible changes in genes not included in the clinical exome panel were thus sought, performing WES of the patient’s DNA. The inclusion of a third filtering step based on HPO terms, or including all genes from the MitoCarta database [20] in the narrowing down of genetic-variants discovered by WES, corroborated the presence of the above change but also identified NM_018480.7: c.83T > C (p.Val28Ala) in homozygous fashion in *TMEM126B*, which encodes a transmembrane component of the mitochondrial complex I. Biallelic changes in *TMEM126B* has been related to the mitochondrial complex I deficiency, nuclear type 29 (MIM#618250). However, this last change, classified as -likely benign- according to the VarSome web platform [14, 21] and as -tolerant- by the recently developed MetaDome web server [22, 23] was discarded as disease-causing based on the existence of two homozygotes for c.83T > C, who show no sign of disease (recorded on the gnomAD platform [24]). No changes in mitochondrial DNA were detected. A complete list of genes and variants identified via WES is provided in Additional file 1, which also compiled consideration for discarding other changes such as those identified in *PHKA2* or *SLC3A1.*

### Literature review

Up to now, 14 patients, included two siblings, having a MCT1 deficiency have been reported. Six were with biallelic pathogenic variants all harboured in homozygous fashion and for the remaining, monoallelic nucleotide changes were reported (see Table 2). Mutational spectrum included two duplications, three deletions, three nonsense changes and one missense change [5–7]. Although several cases of symptomatic monoallelic carriers have been described, not all the carriers display symptoms [8, 9], even when the same mutation in involved, suggesting that possible triggers are also needed for the development of the recurrent ketoacidosis.

**Table 2:** Characteristics of the patients with MCT1 deficiency

	van Hasselt, 2014									Al-Khawaga 2019	Nicolas-Jilwan, 2020; 2 siblings		Balasubramaniam, 2015	Le, 2020
Ethnicity	Syrian	Irish	Turkish	British	British	British	British	Dutch	Dutch	nr	nr	nr	British	Sephardic Jewish
Consanguinity	Yes	No	Yes							Yes	Yes	Yes		
Allele 1 (Variant/ Effect)	**c.41dup** (p.Asp15Argfs*34)	**c.937C > T** (p.Arg313*)	**c.982C > T** (p.Arg328*)	c.586C > T (p.Arg196*)	c.747_750del (p.Asn250Serfs*5)	c.747_750del (p.Asn250Serfs*5)	c.499del (p.Val167Phefs*13)	c.490dup (p.Leu164Profs*46)	c.938G > A (p.Arg313Gln)†	**c.218del** (p.Gly73Valfs*8)	**c.1079del** (p.Ala360Glyfs*19)	Sibling of the previous patient (no genetic study performed)	c.982C > T (p.Arg328*)	c.41dup (p.Asp15Argfs*34)
Allele 2 (Variant/ Effect)	**c.41dup** (p.Asp15Argfs*34)	**c.937C > T** (p.Arg313*)	**c.982C > T** (p.Arg328*)	–	–	–	–	–	–	**c.218del** (p.Gly73Valfs*8)	**c.1079del** (p.Ala360Glyfs*19)		–	–
Metabolic derangement	Profound ketoacidosis	Profound ketoacidosis	Profound ketoacidosis	Cyclic vomiting	Ketoacidosis	Ketoacidosis	Ketotic hypoglycemia	Ketoacidosis	Cyclic vomiting	Recurrent attacks of hypoglycemia and metabolic acidosis; massive ketonuria, normal acid lactic	Recurrent ketoacidosis, no hypoglycemia	Recurrent ketoacidosis, hypoglycemia	Recurrent ketoacidosis, no hypoglycemia	Ketotic hypoglycemia on ketogenic diet
Metabolic screening	–	–	–	–	–	–	–	–	–	Normal serum ammonia, lactate, pyruvate, amino acids, and acyl carnitine. Normal urine succinyl acetone, orotic, and organic acids	Normal plasma lactate, and ammonia	–	Normal serum ammonia and lactate. Massive ketonuria, increased urine 2- methyl-3-hydroxybutyrate, 2-methylacetoacetate and tiglylglycine	–
Brain MRI	–	–	–	–	–	–	–	–	–	Heterotopia, white matter diffuse alterations. Normal spectroscopy (no lactate elevation)	White matter diffuse alteration, including basal ganglia, corpus callosum agenesia	White matter diffuse alteration, including basal ganglia, corpus callosum agenesia	–	Normal
Epilepsy	–	Yes	–	–	–	–	–	–	–	Yes	Yes	Generalized tonic–clonic seizures	–	Absent seizures
Psychomotor delay	Moderate intellectual disability	Moderate intellectual disability	Mild intellectual disability	–	–	–	–	–	–	Normal at the age of 18 months	Motor and speech delay	Developmental delay	–	–
Congenital malformations	Atrial septal defect, hypoplastic left pulmonary artery, and main bronchus	–	Cleft palate	–	–	–	–	–	–	–	–	Left kidney agenesia Intrauterine growth retardation	–	–
Others	Microcephaly			Migraine	Migraine			Short stature	Exercise intolerance			Failure to thrive Fatty liver		Microcephaly

The bold corresponds to biallelic pathogenic variants

Variant nomenclature follows the recommendations of Human Genome Variation Society (HGVS) (https://varnomen.hgvs.org/)

The age at diagnosis varied between 3–23 months for the homozygous patients. Clinically these patients present severe ketoacidosis episodes during glucose shortage events such as infections or fasting (see Table 2). During the metabolic crisis, they received treatment with dextrose infusion together with sodium bicarbonate to correct the acidosis [5–7]. Interestingly, although the ketoacidosis crisis may be present at 3 days of life [6], after the age of 7–8 years old there seems to be a complete resolution of the metabolic crisis, which might suggest adaptative mechanisms or environmental factors [5, 7, 8]. The present patient had the last documented ketoacidosis attack at the age of 15 months.

Moreover, the homozygous patients have common central nervous clinical features like psychomotor delay, epilepsy or corpus callosum agenesia, with a distinctive neuroimaging pattern [5–7], differently from other inborn errors of ketone utilization like succinyl CoA oxoacid transferase (SCOT) deficiency (MIM#245050) and mitochondrial acetoacetyl-CoA thiolase (ACAT1) deficiency (MIM#203750) [25].

## Discussion

The details of MCT1 deficiency remain to be understood. To the best of our knowledge there are only six patients with biallelic pathogenic variants causing MCT1 deficiency reported up to now in the literature, see Table 2 [5–7]. These patients all suffered recurrent episodes of severe ketoacidosis during catabolic events such as fasting or infection, especially in their first years of life. Moreover, patients with a monoallelic change in *SLC16A1* also experience recurrent episodes of acidosis, see Table 2 [5, 8, 9], suggesting that monoallelic carriers may be more prevalent than would seem apparent. The present patient suffered several severe episodes of metabolic acidosis due to ketosis, but also had lactate accumulation. Her monoallelic carrier family members experienced no cyclic vomiting or recurrent ketosis, but none underwent any biochemical study. Notwithstanding that the absence of any remarkable symptomatology has previously been reported in family members of other patients with monoallelic pathogenic changes for *SLC16A1* [8], the health status of mutation carriers should be monitored for possible late manifestation of the disease.

Apart from the ketoacidosis attacks, the biallelic variants for MCT1 deficiency patients described so far also present other common features such as important central nervous system involvement including neuroimaging abnormalities, psychomotor delay, epilepsy and agenesis of corpus callosum [5–7]. That might reaveal the consequences that MCT1 deficiency might have on brain energy homeostasis.

MCT1 is responsible for the H^+^-coupled transport of short chain monocarboxylates, primarily L-lactate, pyruvate or ketone bodies that enter the mitochondria as respiratory fuel [26]. Indeed, MCT1 has been described as a major player in whole-body energy homeostasis and the distribution of redox potential between organs [27]. To date, however, lactic acidosis has not been detected in patients with MCT1 deficiency. Recent studies in the Schwann cells of MCT1 knockout mice, have, nonetheless, detected reductions in spare respiratory capacity similar to those seen in the present patient, leading to impaired mitochondrial function [28].

The central nervous system is highly dependent on a continuous energy supply. The human brain accounts for some 20% of the body’s total expenditure at rest [29, 30], while the developing brain consumes 40% [31]. Glucose is the main fuel employed, and glucose transporters ensure its efficient uptake by neural cells. During glucose shortage events, however, such as fasting or catabolic episodes, the brain can metabolize other organic substrates, especially lactic acid and ketone bodies [32]. Monocarboxylate transporters play a crucial role in the use of these substrates [33]. In recent years, a growing body of evidence has revealed lactate to be an energy substrate for the brain as well, sustaining its neuronal activity during glucose deprivation [34–37]. The hyperlactacidemia observed during hypoglycemia episodes in glycogen storage disease type I might be another example [25]. It has also been suggested that monocarboxylates represent substantial energy substrates, especially for the developing brain [38, 39]. For example, lactate is elevated in newborns' blood immediately after delivery, providing an important brain energy substrate for the first few hours after birth [40]. In neonatal mice, brain lactate is successfully utilized in the hippocampus as an energy substrate, and maintains synaptic function [41]. Ketone bodies are also an important source of energy for the brain under certain conditions, including fasting, ketogenic diets, and in breastfed newborn babies [42–44].

Interestingly, it has been recently shown that monocarboxylates might also be involved in more complex metabolic pathways such as myelin synthesis. In rat cerebellum and corpus callosum, white matter oligodendrocytes take up lactate via MCT1 to ensure neuron survival and myelination in low-glucose conditions [45, 46]. Moreover, in animal studies oligodendrocytes progenitor cells involved in myelination utilize lactate for cell cycling and differentiation in metabolic pathways in which MCT1 is a central figure. This has important implications in high myelin brain regions such as the corpus callosum [47, 48].

From this point of view, the MCT1 deficiency induces impaired monocarboxylate transport and might be involved in important energetic perturbations in nervous system energy metabolism that become more evident during glucose shortage events and could affect important processes such as myelination in the developing brain. That may explain the presence of MRI findings in high myelin brain regions like basal ganglia or corpus callosum and clinical features such as psychomotor delay or epilepsy. This hypothesis is supported by the present bioenergetic analysis performed with the patient’s fibroblasts. Here, the maximal mitochondrial oxygen consumption capacity following the addition of FCCP, which mimics a physiologic energy demand, was significantly decreased, confirming an impaired capacity for adapting to stress. The remodelling seen in mitochondrial size and the appearance of the cristae might be the response to the energetic challenge posed, as changes in cellular metabolic demands or homeostatic insults are known to correlate with changes in the mitochondrial ultrastructure [49]. It remains to be clarified whether other genetic variations influence mitochondrial fitness to adapt.

In summary, while MCT1 deficiency is associated with recurrent episodes of ketoacidosis, it may also involve episodes of lactic acidosis, neuromotor delay and neuroimaging anomalies of the subcortical white matter and basal ganglia or agenesis of the corpus callosum. The multiple pathways that monocarboxylates might follow in the neurons and glia, such as the production of ATP or myelin synthesis, might explain the complex features associated with biallelic MCT1 deficiency. MCT1 phenotype is probably the result of a plethora of causes including a dosage effect of the *SLC16A1* variants, but also the result of other contributors such as combinations of cis or trans-acting changes that should be relevant for the global response to an energetic shortage.

## Supplementary Information

**Additional file 1:** A complete list of genes and variants identified via WES.

## Data Availability

All data generated or analyzed during this study are included in this published article and its supplementary information files.
