# Supplementary material for: Mitochondrial bioenergetic is impaired in Monocarboxylate transporter 1 deficiency: a new clinical case and review of the literature
Source: Orphanet J Rare Dis. 2022 Jun 21;17:243. doi: 10.1186/s13023-022-02389-4 (PMC9215049; doi:10.1186/s13023-022-02389-4)
Supplement: Supplementary file 1 — Additional file 1: A complete list of genes and variants identified via WES. [file 13023_2022_2389_MOESM1_ESM.docx]

**Supplementary Table 1.** For analysis of the genetic variants obtained via WES, those with a MAF of <0.005 were filtered and those that cause a loss of function prioritized. Screening was performed using virtual panels based on the phenotypic importance according to human phenotype ontology (HPO) terms or the MitoCarta 2.0 database. Variants that might influence patient's phenotype are highlighted in grey. Information about platforms gnomAD, SIFT, Polyphen2, ClinVar, Varsome (ACMG classification) are listed. Other relevant information taken into consideration to discard the variants is detailed in the last column, based on OMIM (https://www.omim.org/), Varsome (https://varsome.com/), and gnomAD (https://gnomad.broadinstitute.org/).

| **Chr** | **Gene** | **Genomic coordinate** | **Type** | **Consequence** | **Genotype** | **HGVS cDNA** | **HGVS protein** | **Allele Freq %**  **(gnomAD)** | **SIFT** | **PolyPhen2** | **ClinVar Significance** | **ACMG classification** | **Other relevant information** |
| --- | --- | --- | --- | --- | --- | --- | --- | --- | --- | --- | --- | --- | --- |
| 1 | *MECR* | 29527085 | snv | missense_variant | het | NM_016011.2:  c.773G>T | NP_057095.2:  p.Arg258Leu | 0.0967 | deleterious (0.02) | benign (0.08) | Benign | VUS | AR inheritance Homozygotes in healthy population |
| 1 | *SLC16A1* | 113460277 | deletion | frameshift_variant | hom | NM_003051.3:  c.747_750del | NP_003042.3:  p.Asn250Serfs*5 | 0.000795 |  |  | Pathogenic | Pathogenic |  |
| 1 | *OBSCN* | 228469903 | mnp | missense_variant,splice_region_variant | het | NM_001271223.2:  c.9754_9755delinsTT | NP_001258152.2:  p.Arg3252Leu | 0 |  | possibly damaging (0.763) | - | VUS | Two individual SNP with high frequency |
| 2 | *PTCD3* | 86364634 | deletion | frameshift_variant | het | NM_017952.5:  c.2025_2028del | NP_060422.4:  p.Asp676Alafs*35 | 0.00477 |  |  | - | Likely pathogenic | AR inheritance |
| 2 | *ABCB6* | 220075474 | snv | downstream_gene_variant | het |  |  | 0 |  |  |  |  | Downstream variant |
| 3 | *CCDC58* | 122081881 | deletion | splice_region_variant,intron_variant | het | NM_001017928.2:  c.325-8del |  | 0.677 |  |  | - | Likely benign | Homozygotes in healthy population |
| 3 | *BDH1* | 197241187 | snv | missense_variant | het | NM_203315.2:  c.510G>C | NP_976060.1:  p.Trp170Cys | 0.000796 | deleterious (0) | probably damaging (0.999) | - | VUS | No associated disease |
| 6 | *ME1* | 83933558 | snv | missense_variant | het | NM_002395.4:  c.1370T>G | NP_002386.1:  p.Val457Gly | 0.00518 | deleterious (0) | probably damaging (0.992) | - | VUS | No associated disease |
| 6 | *TRMT11* | 126319252 | snv | missense_variant | het | NM_001031712.2:  c.266C>G | NP_001026882.2:  p.Ser89Cys | 0.00119 | deleterious (0) | benign (0.071) | - | VUS | No associated disease |
| 11 | *TMEM126B* | 85342732 | snv | missense_variant,splice_region_variant | hom | NM_018480.4:  c.83T>C | NP_060950.3:  p.Val28Ala | 0.0639 | deleterious - low confidence (0.03) | benign (0.005) | Likely benign | Likely benign | Homozygotes in healthy population |
| 13 | *VWA8* | 42465596 | snv | missense_variant | het | NM_015058.1:  c.611G>A | NP_055873.1:  p.Arg204His | 0.00278 | deleterious (0) | probably damaging (0.999) | - | VUS | No associated disease |
| 14 | *SLC25A21* | 37180615 | snv | missense_variant | het | NM_030631.3:  c.511G>A | NP_085134.1:  p.Gly171Arg | 0.0971 | deleterious (0) | probably damaging (1) | - | VUS | AR inheritance Homozygotes in healthy population |
| 17 | *FDXR* | 72860347 | snv | stop_gained | het | NM_001258012.1:  c.1054C>T | NP_001244941.1:  p.Arg352Ter | 0.00413 |  |  | Likely pathogenic | Pathogenic | AR inheritance |
| 19 | *SLC25A41* | 6426528 | snv | missense_variant | het | NM_173637.3:  c.985C>T | NP_775908.2:  p.Arg329Trp | 0.00402 | deleterious (0.05) | benign (0.011) | - | VUS | No associated disease |
| 22 | *CHCHD10* | 24108461 | snv | missense_variant,splice_region_variant | het | NM_213720.1:  c.263C>G | NP_998885.1:  p.Ala88Gly | 0 | tolerated (0.3) | benign (0.304) | - | VUS | Most pathogenicity predictors: benign |

1. Gene list based on MitoCarta 2.0

ACMG: American College of Medical Genetics; HGVS: Human Genome Variation Society

1. Gene list based on HPO

| **Chr** | **Gene** | **Genomic coordinate** | **Type** | **Consequence** | **Genotype** | **HGVS cDNA** | **HGVS protein** | **Allele Freq %**  **(gnomAD)** | **SIFT** | **PolyPhen2** | **ClinVar Significance** | **ACMG classification** | **Other relevant information** |
| --- | --- | --- | --- | --- | --- | --- | --- | --- | --- | --- | --- | --- | --- |
| 1 | SLC16A1 | 113460277 | deletion | frameshift_variant | hom | NM_003051.3:  c.747_750del | NP_003042.3:  p.Asn250Serfs*5 | 0 |  |  | Pathogenic | Pathogenic |  |
| 1 | KCNJ10 | 160011671 | snv | missense_variant | het | NM_002241.4:  c.652C>T | NP_002232.2:  p.Leu218Phe | 0.00159 | deleterious (0.04) | probably damaging (0.914) | VUS | Likely pathogenic | AR inheritance |
| 2 | SLC3A1 | 44507992 | snv | missense_variant | het | NM_000341.3:  c.568A>G | NP_000332.2:  p.Met190Val | 0.00437 | deleterious (0) | probably damaging (0.952) | - | Likely pathogenic | AR inheritance  No biochemical markers found |
| 2 | ALMS1 | 73680645 | snv | missense_variant | het | NM_015120.4:  c.6988T>C | NP_055935.4:  p.Cys2330Arg | 0 |  | probably damaging (0.966) | VUS | VUS | AR inheritance |
| 2 | ERBB4 | 212587119 | snv | splice_region_variant,synonymous_variant | het | NM_005235.2:  c.882A>G | NM_005235.2:  c.882A>G(p.=) | 0.427 |  |  | Likely benign | Benign | Homozygotes in healthy population |
| 3 | FLNB | 58095866 | snv | missense_variant | het | NM_001164317.1:  c.2453G>A | NP_001157789.1:  p.Arg818Gln | 0.0903 | tolerated (0.29) | possibly damaging (0.883) | Conflicting interpretations of Pathogenicity | VUS | Homozygotes in healthy population |
| 4 | SLC34A2 | 25667747 | snv | splice_region_variant,intron_variant | het | NM_006424.2:  c.380-3C>T |  | 0.0119 |  |  |  | Likely benign | Splicing predictors: benign |
| 4 | FAT4 | 126238305 | snv | missense_variant | het | NM_024582.4:  c.739C>A | NP_078858.4:  p.Pro247Thr | 0.421 |  | probably damaging (0.999) | Conflicting interpretations of Pathogenicity | VUS | Homozygotes in healthy population |
| 6 | CUL7 | 43006418 | deletion | frameshift_variant | het | NM_001168370.1:  c.4703_4704del | NP_001161842.1:  p.Val1568Glyfs*69 | 0 |  |  | Pathogenic | Likely pathogenic | AR inheritance |
| 6 | DST | 56425092 | snv | missense_variant,splice_region_variant | het | NM_001144769.2:  c.8083G>A | NP_001138241.1:  p.Asp2695Asn | 0.039 |  | probably damaging (0.967) | Likely benign | Pathogenic | AR inheritance Most pathogenicity predictors: benign |
| 6 | DLL1 | 170592605 | snv | missense_variant | het | NM_005618.3:  c.1762G>A | NP_005609.3:  p.Glu588Lys | 0.00601 | deleterious (0.03) | benign (0.048) | - | Benign |  |
| 7 | GLI3 | 42063171 | snv | missense_variant | het | NM_000168.5:  c.1393G>C | NP_000159.3:  p.Gly465Arg | 0.393 | tolerated (0.07) | probably damaging (0.935) | Likely benign | Benign | Homozygotes in healthy population |
| 11 | LRP4 | 46894654 | snv | missense_variant | het | NM_002334.3:  c.4580G>A | NP_002325.2:  p.Arg1527His | 0.00119 | deleterious (0.01) | probably damaging (0.98) | - | VUS | Different phenotype association |
| 11 | TMEM126B | 85342732 | snv | missense_variant,splice_region_variant | hom | NM_018480.4:  c.83T>C | NP_060950.3:  p.Val28Ala | 0.4 | deleterious - low confidence (0.03) | benign (0.005) |  | Likely benign | Homozygotes in healthy population |
| 12 | MYO1H | 109877467 | snv | missense_variant | het | NM_001101421.3:  c.2278C>T | NP_001094891.3:  p.Pro760Ser | 0.0447 | deleterious (0.03) | benign (0.423) | - | VUS | Most pathogenicity predictors: benign |
| 12 | MYO1H | 109882051 | deletion | frameshift_variant | het | NM_001101421.3:  c.2812_2815del | NP_001094891.3:  p.Val938Phefs*57 | 0.0012 |  |  | - | Likely pathogenic | Different phenotype association |
| 16 | ABCA3 | 2336870 | snv | missense_variant | het | NM_001089.2:  c.3103C>T | NP_001080.2:  p.Arg1035Cys | 0.00318 | deleterious (0.03) | benign (0.041) | - | VUS | Inheritance AR  Most pathogenicity predictors: benign |
| 17 | SOX9 | 70119713 | snv | missense_variant | het | NM_000346.3:  c.715A>C | NP_000337.1:  p.Thr239Pro | 0.00214 | deleterious (0.01) | probably damaging (0.996) | Likely benign | Likely pathogenic | Different phenotype association |
| 22 | CHCHD10 | 24108461 | snv | missense_variant,splice_region_variant | het | NM_213720.1:  c.263C>G | NP_998885.1:  p.Ala88Gly | 0 | tolerated (0.3) | benign (0.304) | - | VUS | Most pathogenicity predictors: benign |
| X | PHKA2 | 18954183 | snv | missense_variant | het | NM_000292.2:  c.1127C>T | NP_000283.1:  p.Pro376Leu | 0.000603 | deleterious (0.03) | possibly damaging (0.828) | VUS | Benign | Hemizygotes in healthy population |

ACMG: American College of Medical Genetics; HGVS: Human Genome Variation Society
